# Genetic variability of *Plasmodium vivax* from primary–relapses paired samples

**DOI:** 10.1186/1475-2875-11-S1-P140

**Published:** 2012-11-09

**Authors:** Flávia C Araujo, Filipe F Andrade, Antônio M Rezende, Cor JF Fontes, Luzia H Carvalho, Cristiana FA Brito

**Affiliations:** 1 Laboratory of Malaria, Research Institute Rene Rachou/Fiocruz, Belo Horizonte, MG, 30190 002, Brazil; 2 Universitary hospital Julio Muller, UFMT, Cuiabá, MT, Brazil

## Background

Human malaria is caused by protozoa from the genus *Plasmodium.* Approximately 40% of world population is at risk of infection. *Plasmodium vivax* is the most worldwide distributed human *Plasmodium* species. In Brazil, more than 300,000 cases of the disease were recorded in 2010 and 86% were caused by *Plasmodium vivax*. *P. vivax* infections are characterized by hepatic dormant forms, the hypnozoites and those are activated in varying intervals of time resulting in the typical relapses. Little is known about the mechanism of latency and activation of hypnozoites associated to relapses. The scarcity of genetic markers for *P. vivax* has hampered the analysis of important parasite phenotypes, such as patterns of relapse. In this context, the aim of this work was to study the variability of the parasites of primary infections and relapses from the same patients.

## Materials and methods

Primary-Relapse paired samples of 30 patients were genotyped using 10 molecular markers (8 microsatellites and blocks 2 and 10 of MSP-1) by capillary electrophoresis on an automated DNA sequencer. Moreover, the presence of multiple infections was confirmed by cloning of amplicons and genotyping of different colonies (mean of 10 colonies per PCR product).

## Results

The majority of parasites showed distinct haplotypes in relapses compared to primary infection. It was demonstrated a high frequency of multiple-clone infections both in primary infection and relapse. A variation of predominant alleles among distinct markers in different malaria recidives of the same individual was observed. Therefore, haplotypes in relapse could also be identified in primary infections as a rare alleles.

## Conclusions

Altogether our findings suggest that mechanisms involved in hypnozoites activation might not be based only on parasites genetic programming but also on host/enviroment factors.

